# Supplementary material for: Optogenetic control of Bacillus subtilis gene expression
Source: Nat Commun. 2019 Jul 15;10:3099. doi: 10.1038/s41467-019-10906-6 (PMC6629627; doi:10.1038/s41467-019-10906-6)
Supplement: Supplementary file 1 — Supplementary Information [file 41467_2019_10906_MOESM1_ESM.pdf]

Supplementary Information for

Optogenetic control of *Bacillus subtilis* gene expression

Sebastian M. Castillo-Hair, Elliot A. Baerman, Masaya Fujita, Oleg A. Igoshin, Jeffrey J. Tabor

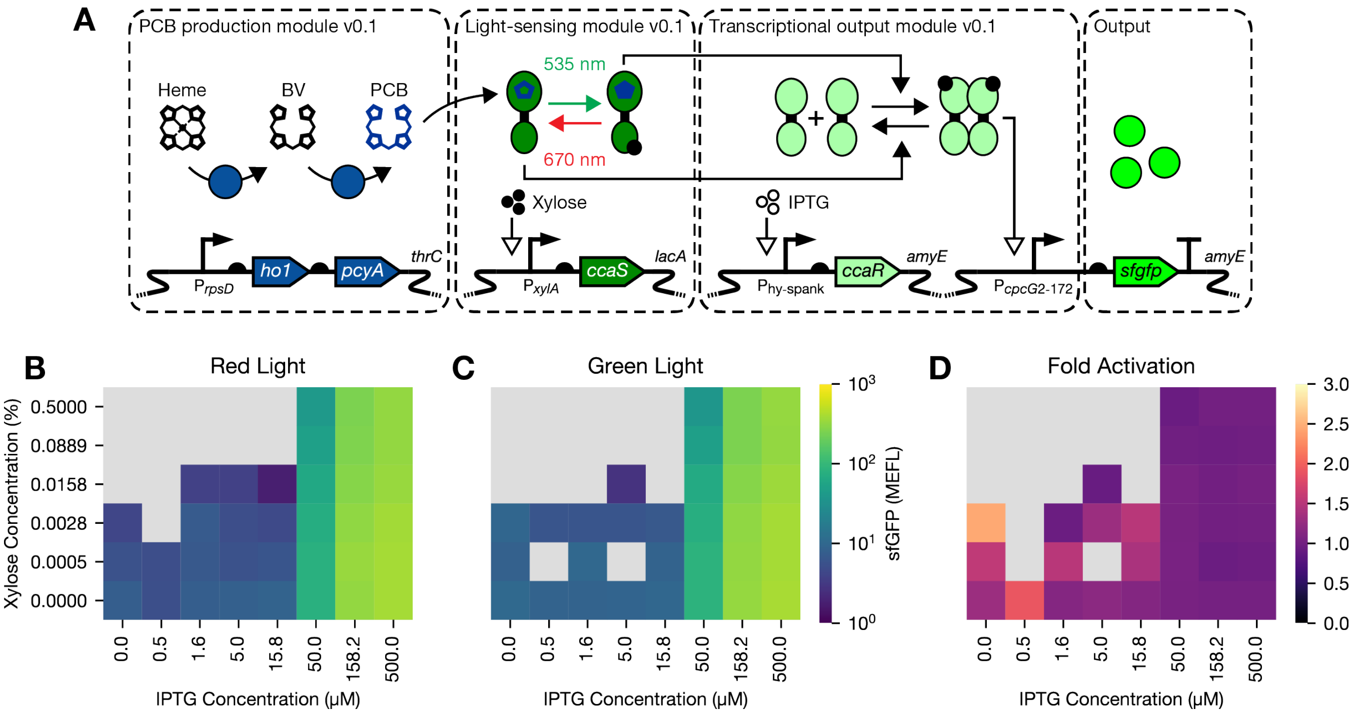

Supplementary Figure 1. Characterization of *B. subtilis* CcaSR v0.1. (A) Genetic device schematic. **(B and C)** sfGFP fluorescence of bacteria expressing CcaSR v0.1 and grown in the presence of different concentrations of IPTG and Xylose and saturating intensities of **(B)** red or **(C)** green light. **(D)** Fold increase in sfGFP fluorescence in green light compared to red light at each xylose and IPTG condition. Fluorescence datapoints show the mean of three experiments run on separate days. Gray squares represent non-detected (N.D) fluorescence values (**Methods**), and corresponding conditions for which fold change was not calculated.

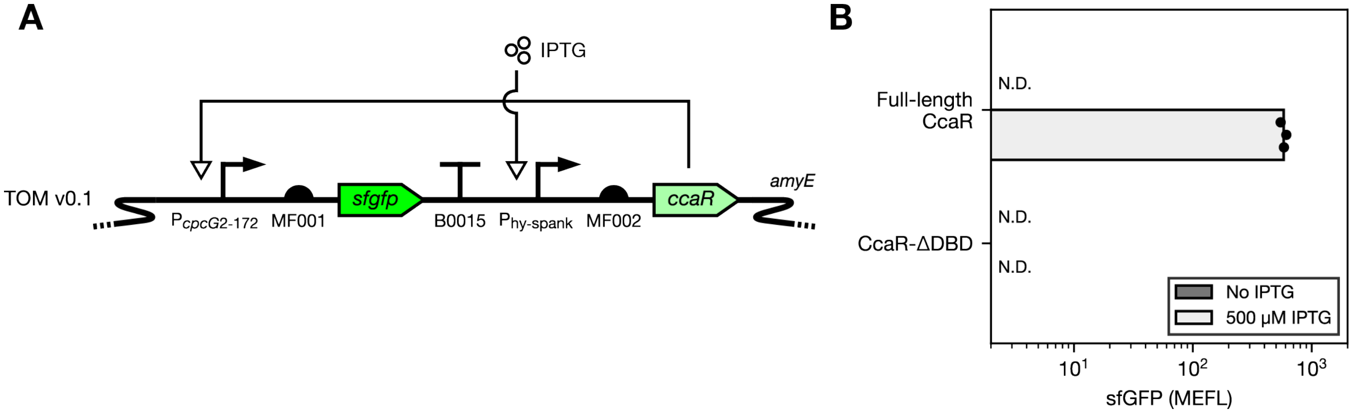

Supplementary Figure 2. sfGFP expression from $P_{cpcG2\text{-}172}$ with overexpression of CcaR and CcaR without its DNA-Binding Domain (CcaR-ΔDBD). (A) Schematic of the Transcription Module and the expected interactions in response to IPTG addition. **(B)** Measured sfGFP in response to IPTG, when either full-length CcaR (top) or a truncated version that lacks the DBD are expressed from $P_{hy\text{-}spank}$. Bars show the mean of three experiments run on separate days. Dots show values of individual experimental replicates. N.D.: not detected (**Methods**).

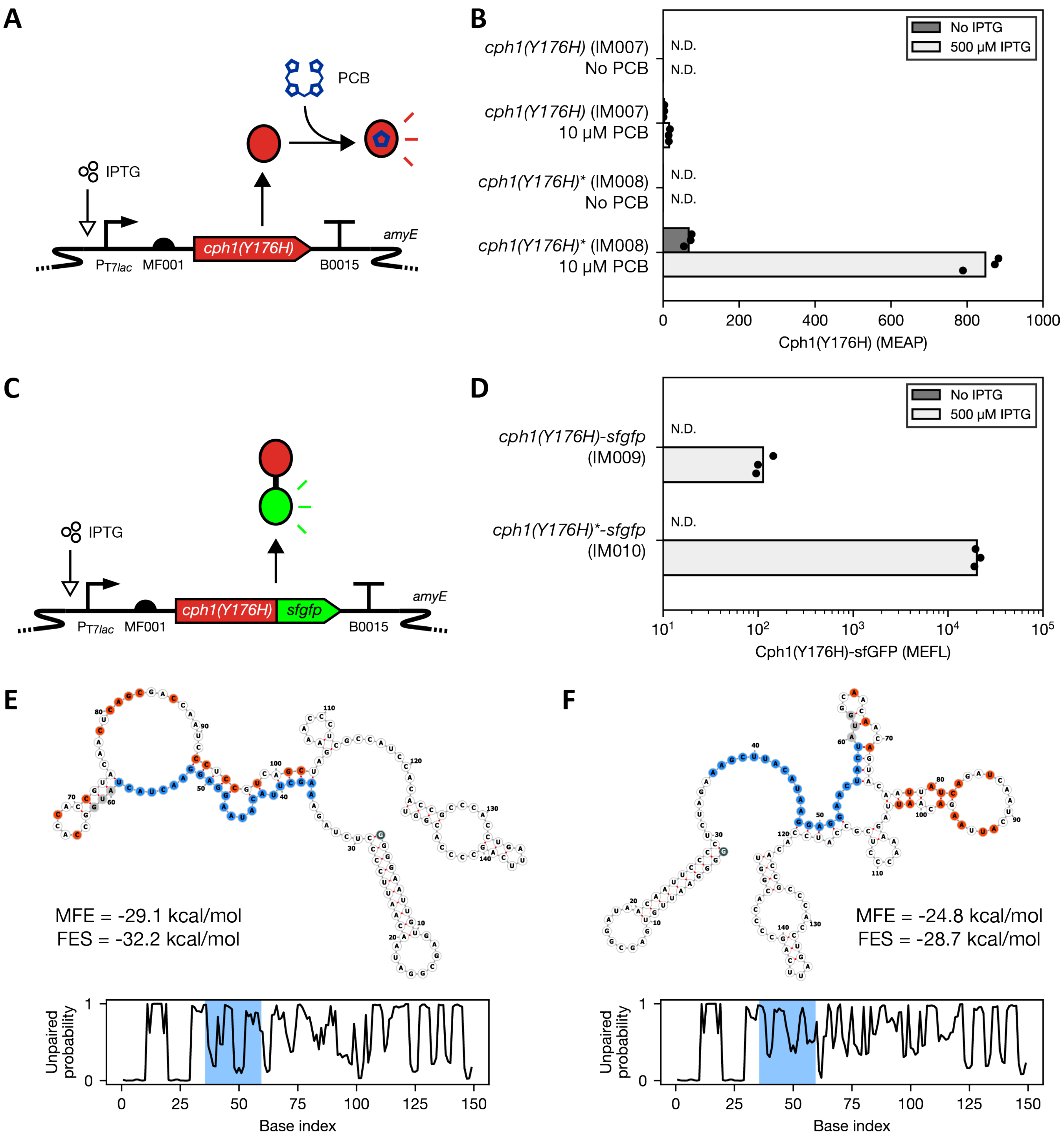

Supplementary Figure 3. Optimization and validation of Cph1(Y176H) for PCB detection. (A) Schematic of *cph1(Y176H)* expressed from the LacI-T7 system[1], and the expected interactions to produce PCB-dependent fluorescence. **(B)** Measured red fluorescence in the presence of zero or 10 µM purified

PCB, when expressing the original *cph1(Y176H)* sequence or one in which the first 15 codons were optimized for reduced mRNA secondary structure (*cph1(Y176H)**) **(C)** Schematic of *cph1(Y176H)-sfgfp* expressed from the LacI-T7 system. **(D)** Measured sfGFP fluorescence in response to IPTG, when expressing *cph1(Y176H)-sfgfp* or *cph1(Y176H)*-sfgfp*. Bars show the mean of three experiments run on separate days. Dots show values of individual experimental replicates. N.D.: not detected (**Methods**). (**E** and **F**) Secondary structure analysis of the *cph1(Y176H*) mRNA before **(E)** and after **(F)** codon optimization of its first 15 codons. Top: Minimum free energy (MFE) secondary structure of the mRNA from the 5' end to the 90th base of the *cph1(Y176H*) ORF. The 5' end is highlighted in dark gray, the RBS sequence in blue, the translation start codon ATG in light grey, and the bases that differ between the original and optimized sequences in orange. MFE and free energy of strand (FES) values are shown. Bottom: probability of finding each base in an unpaired configuration. The RBS region is shown shaded in blue.

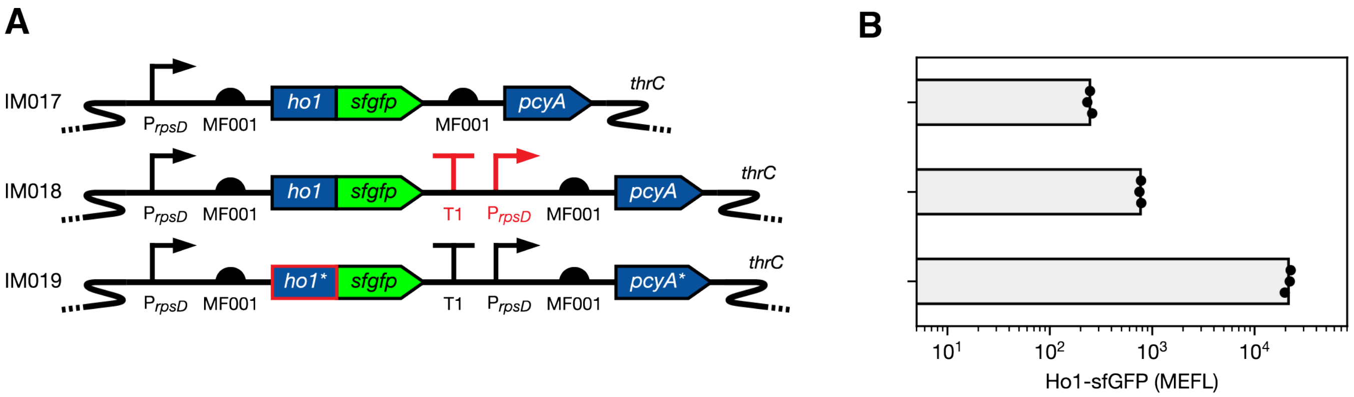

Supplementary Figure 4. Measurement and optimization of *ho1* expression. (A) Schematics of sequentially modified PPMs, wherein *ho1* has been tagged with *sfgfp*. IM17 is equivalent to PPM v0.1, with the addition of the *sfgfp* tag. Similarly, the *ho1* expression cassette in IM019 is equivalent to that of PPM v0.2. Each sequential modification is highlighted in red. **(B)** Measured Ho1-sfGFP fluorescence. The most dramatic increase is observed after codon-optimization of the initial *ho1* sequence (from 769 ± 17 to 21,400 ± 1,500 MEFL). Bars show the mean of three experiments run on separate days. Dots show values of individual experimental replicates.

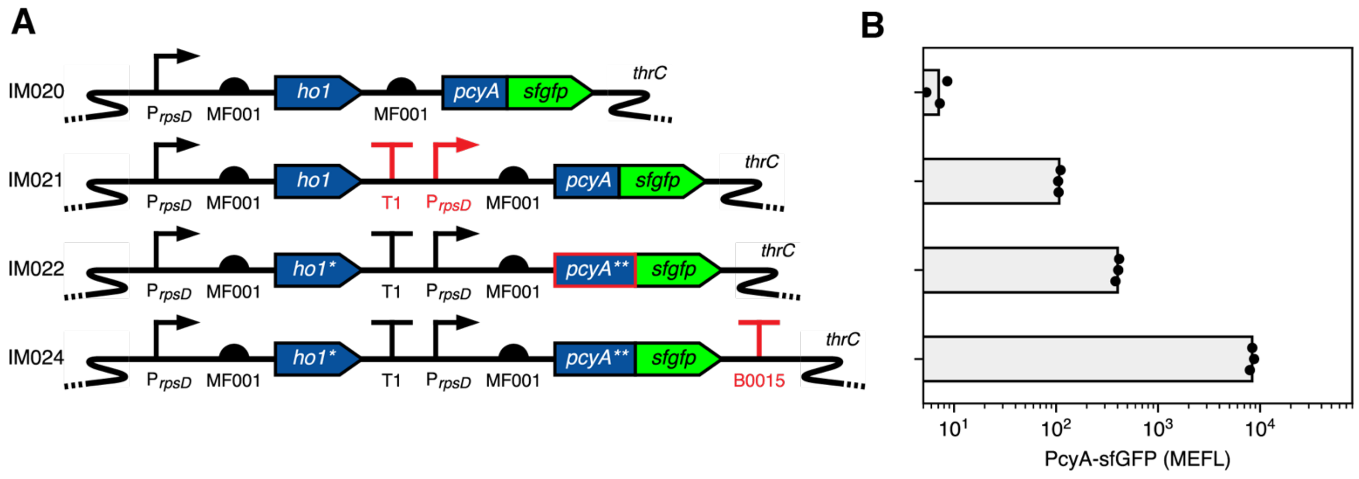

Supplementary Figure 5. Measurement and optimization of *pcyA* expression. (A) Schematics of sequentially modified PPMs, wherein *pcyA* has been tagged with *sfgfp*. IM20 and IM24 are equivalent to PPM v0.1 and v0.2, respectively, with the addition of the *sfgfp* tag. Each sequential modification is highlighted in red. **(B)** Measured PcyA-sfGFP fluorescence. Starting from a very low value (7.1 ± 1.6 MEFL), fluorescence increases dramatically after separation into its own transcriptional unit (107.2 ± 3.1 MEFL), full codon-optimization of *pcyA* (402 ± 17 MEFL), and the addition of a transcriptional terminator (8350 ± 400 MEFL). The large effect of a transcriptional terminator in expression is also observed in a different chromosomal locus (**Supplementary Figure 7**). Bars show the mean of three experiments run on separate days. Dots show values of individual experimental replicates.

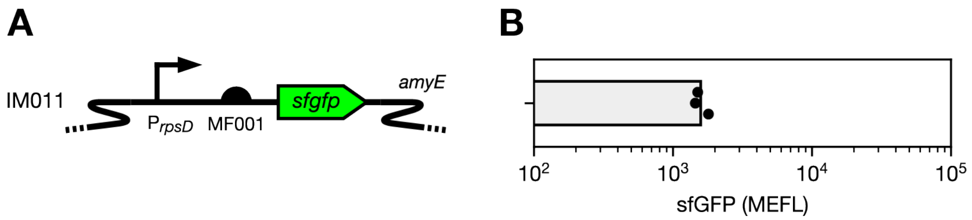

Supplementary Figure 6. sfGFP expression from constitutive promoter P_{rpsD}. (A). Schematic of *sfgfp* expressed from P_{rpsD} and synthetic RBS MF001. **(B)** Measured sfGFP fluorescence. Bars show the mean of three experiments run on separate days. Dots show values of individual experimental replicates.

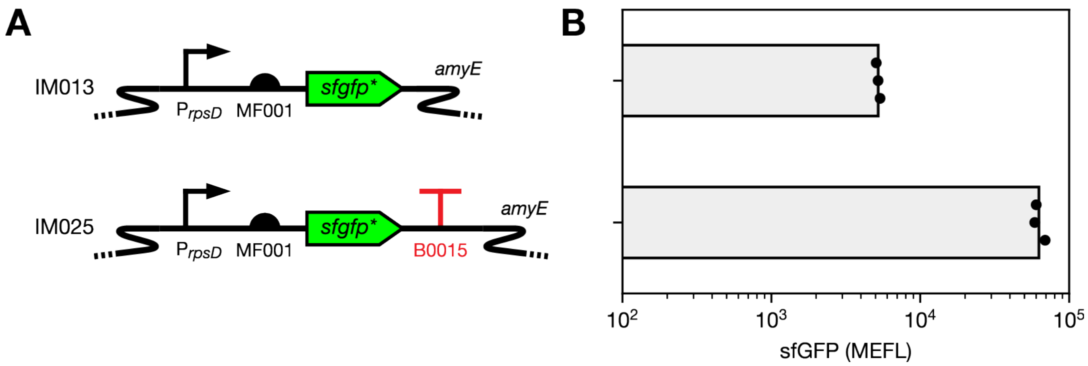

Supplementary Figure 7. A transcriptional terminator dramatically increases gene expression from the *amyE* locus. (A) Genetic device schematics of cassette wherein *sfgfp* is expressed from P_{rpsD} without or with a terminator. **(B)** Measured sfGFP fluorescence. Bars show the mean of three experiments run on separate days. Dots show values of individual experimental replicates.

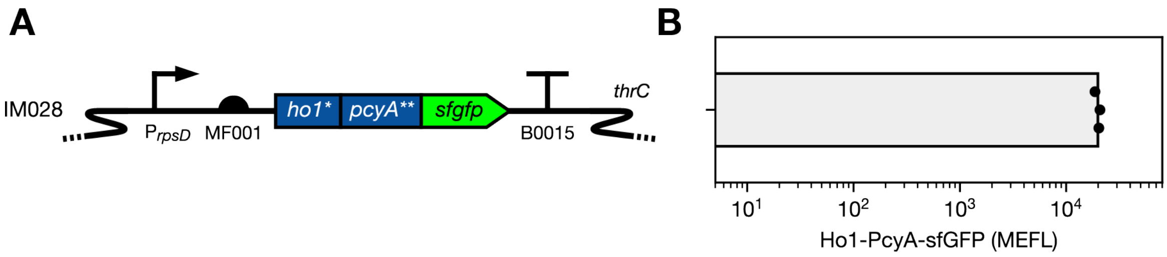

Supplementary Figure 8. Expression of fused *ho1-pcyA* in PPM v0.3 is similar to expression of *ho1* and *pcyA* in PPM v0.2. **(A)** Schematic of a genetic device equivalent to PPM v0.3, with *sfgfp* fused to the C-terminus of chimeric enzyme *ho1-pcyA*. **(B)** Measured sfGFP fluorescence. Compare to fluorescence levels in **Supplementary Figure 4** and **Supplementary Figure 5**. Bars show the mean of three experiments run on separate days. Dots show values of individual experimental replicates.

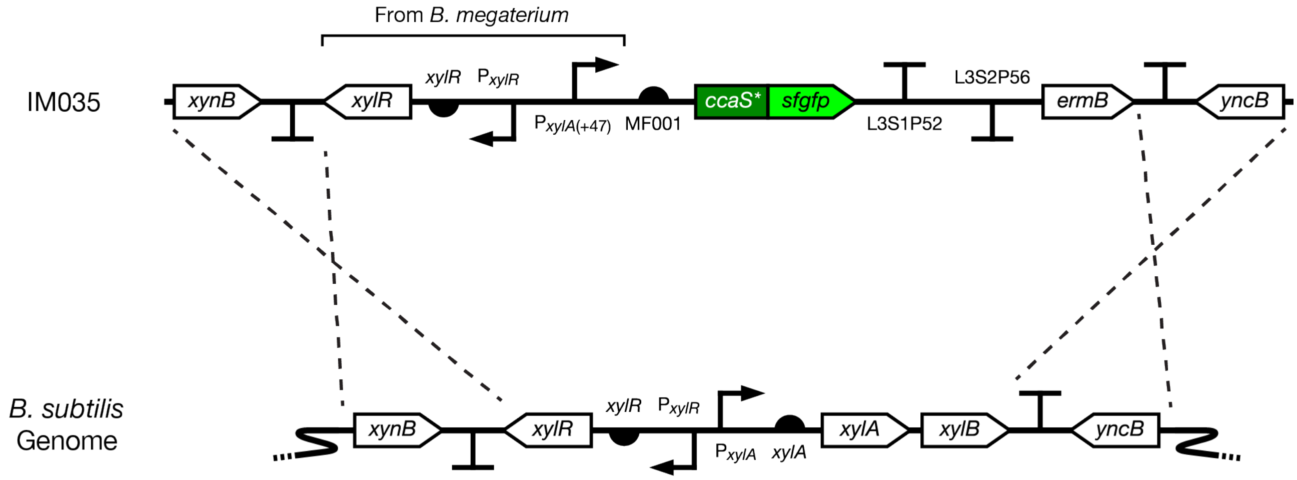

Supplementary Figure 9. Chromosomal integration of CcaS-expressing cassette IM035 into the *xylA* locus. Top: Sequence diagram of IM035 (**Figure 3**), equivalent to LSM 0.2 but with *sfgfp* fused to the C-terminus of *ccaS**. Bottom: schematic of the *xylA* locus in the *B. subtilis* genome. Dashed lines indicate regions expected to engage in homologous recombination.

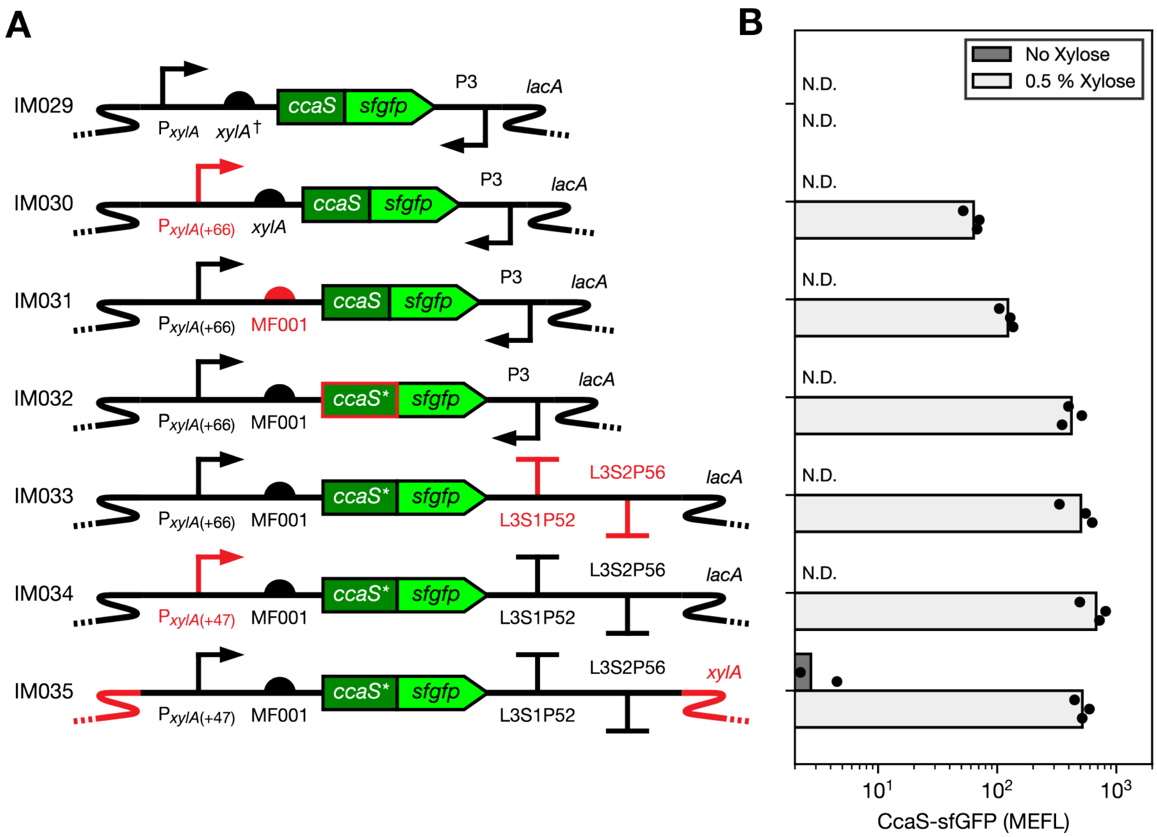

Supplementary Figure 10. Effects of the individual LSM modifications from Figure 3 on *ccaS* expression. (A) Diagrams of sequentially modified LSMs, wherein *ccaS* has been tagged with *sfgfp*. Each sequential modification is highlighted in red. IM29 and IM35 are equivalent to LSM v0.1 and v0.2, respectively, with the addition of the *sfgfp* tag. **(B)** Measured sfGFP fluorescence of each LSM variant in the absence or presence of xylose. Bars show the mean of three experiments run on separate days. Dots show values of individual experimental replicates. N.D.: not detected (**Methods**).

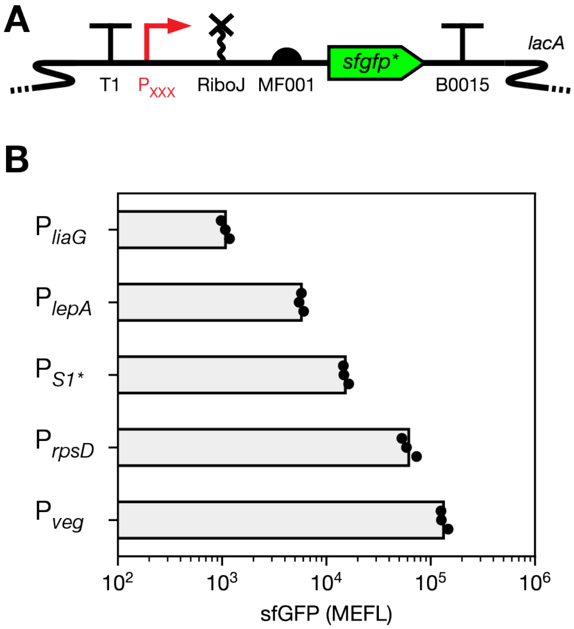

Supplementary Figure 11. Constitutive promoter library used as a promoter activity reference for $P_{cpcG2\text{-}172}$. (A) Schematic of a promoter characterization cassette on which a promoter's transcriptional strength can be measured. Here, a promoter under evaluation is flanked by terminator T1 upstream, and RiboJ, RBS MF001, a *sfgfp* sequence with a codon-optimized N-terminus, and terminator B0015 downstream. Terminator T1 should interrupt any upstream transcription. RiboJ standardizes the resulting mRNA by cleaving the promoter-dependent 5'UTR, making translation independent of the promoter. Therefore, any difference in sfGFP fluorescence should be a function only of the promoter's transcription strength. **(B)** sfGFP fluorescence from cassettes such as in the diagram above, with promoters P_{liaG}, P_{lepA}, P_{S1*}, P_{rpsD}, and P_{veg}. Bars show the mean of three experiments run on separate days. Dots show values of individual experimental replicates.

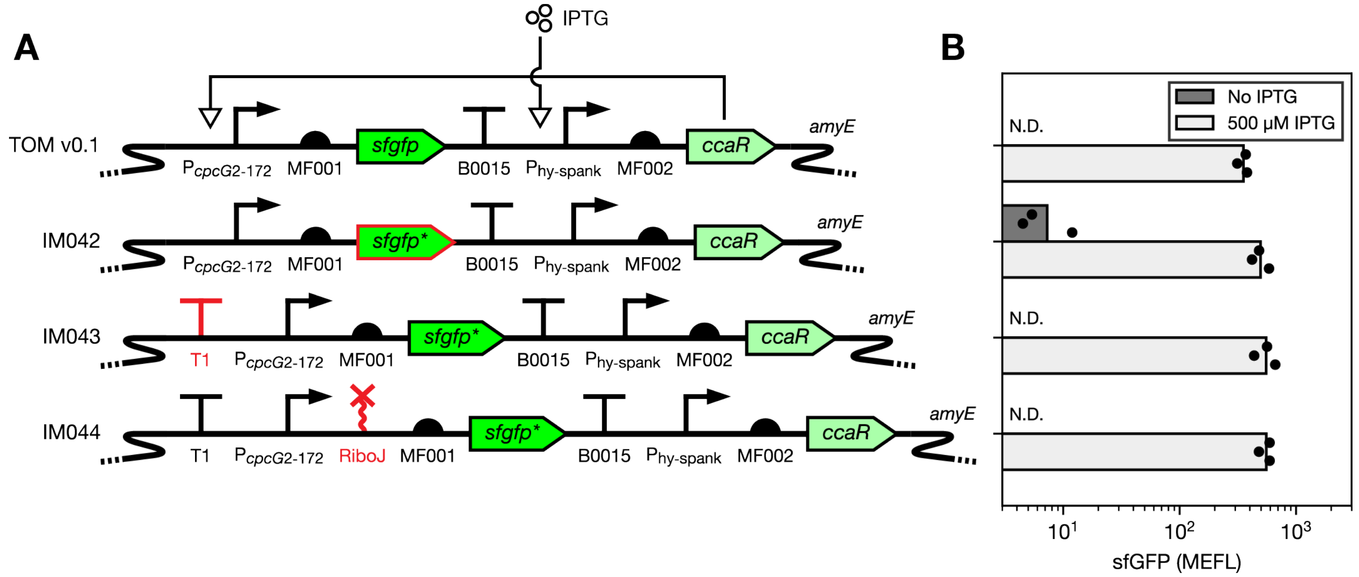

Supplementary Figure 12. Use of the promoter characterization cassette to measure P*cpcG2*-172 activity in the context of the TOM. We start from the TOM v0.1, and sequentially introduce the following changes: codon-optimization of the initial sequence of *sfgfp*, addition of a terminator upstream of P*cpcG2*-172, and addition of RiboJ. These modifications place P*cpcG2*-172 in a promoter characterization cassette equivalent to the one in **Supplementary Figure 11**. **(A)** Diagrams of sequentially modified Transcription Modules, with each modification highlighted in red. **(B)** Measured fluorescence in the absence or presence of saturating IPTG. Bars show the mean of three experiments run on separate days. Dots show values of individual experimental replicates. N.D.: not detected (**Methods**).

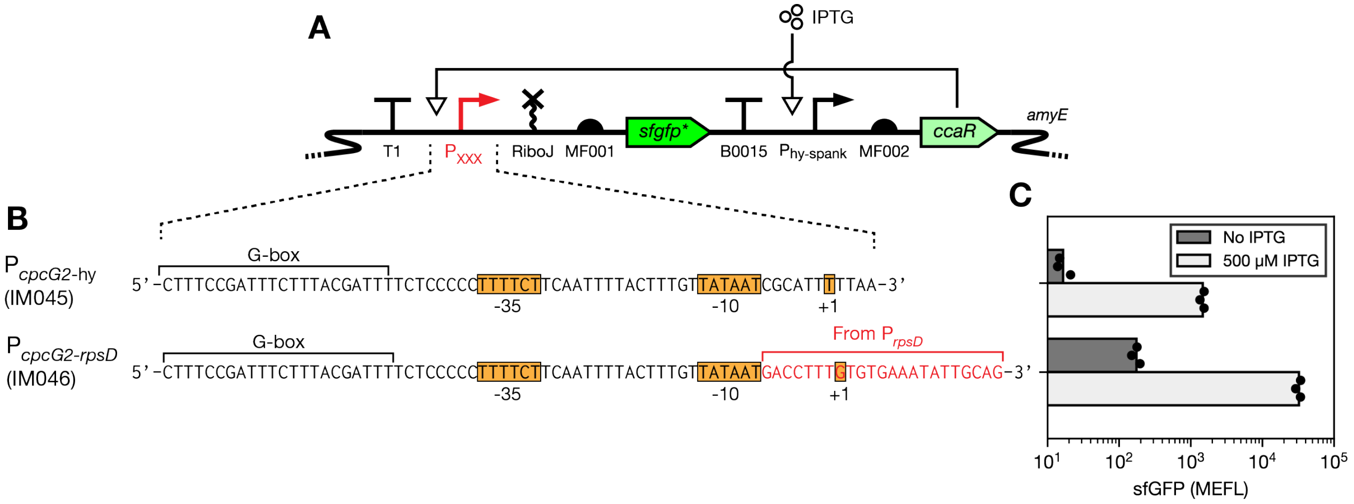

Supplementary Figure 13. Strong transcription is obtained from a chimeric P*cpcG2-rpsD* promoter. (A) Characterization of P*cpcG2*-derived promoter variants using a standard promoter characterization cassette, as in **Figure 4**. **(B)** Sequence of P*cpcG2*-derived variants P*cpcG2*-hy which has a consensus -10 hexamer (**Figure 4**), and P*cpcG2-rpsD* where the sequence after the -10 hexamer was replaced with the sequence from P*rpsD*. **(C)** sfGFP fluorescence resulting from each output promoter system shown in panel (**B**) in the absence and presence of saturating IPTG. Bars show the mean of three experiments run on separate days. Dots show values of individual experimental replicates.

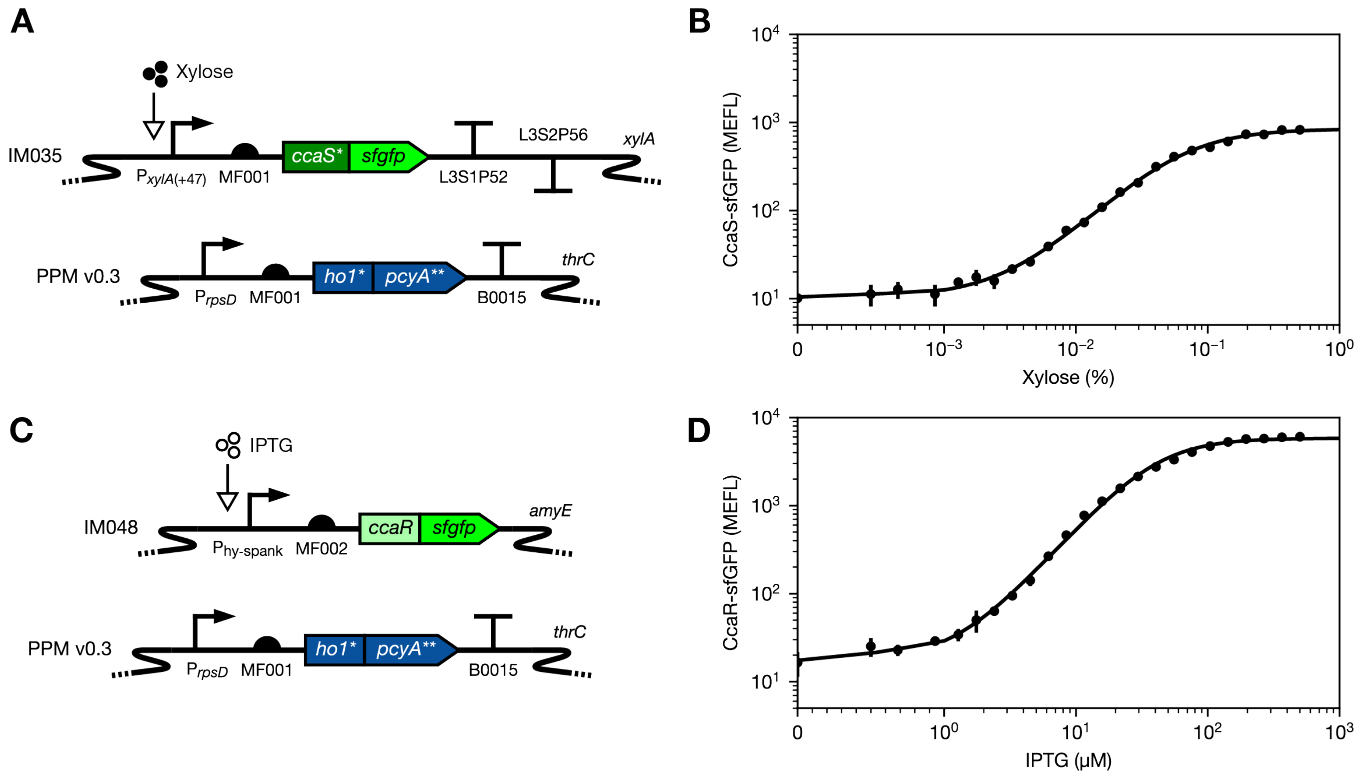

Supplementary Figure 14. Expression curves of CcaS and CcaR used to calculate inducer concentrations in Supplementary Figure 16. (A) Schematics of integration modules used to measure xylose-dependent CcaS expression in a PCB-producing strain. IM035 is equivalent to LSM v0.2 with *sfgfp* fused to the C-terminus of *ccaS*. **(B)** Measured CcaS-sfGFP fluorescence in response to xylose. **(C)** Schematics of integration modules used to measure IPTG-dependent CcaR expression in a PCB-producing strain. IM048 is equivalent to TOM v0.2 with *sfgfp* fused to the C-terminus of *ccaR*. **(D)** Measured CcaR-sfGFP fluorescence in response to IPTG. Markers and error bars show the mean and standard deviation of three experiments run on separate days. Black lines represent Hill Function model fits (**Methods**).

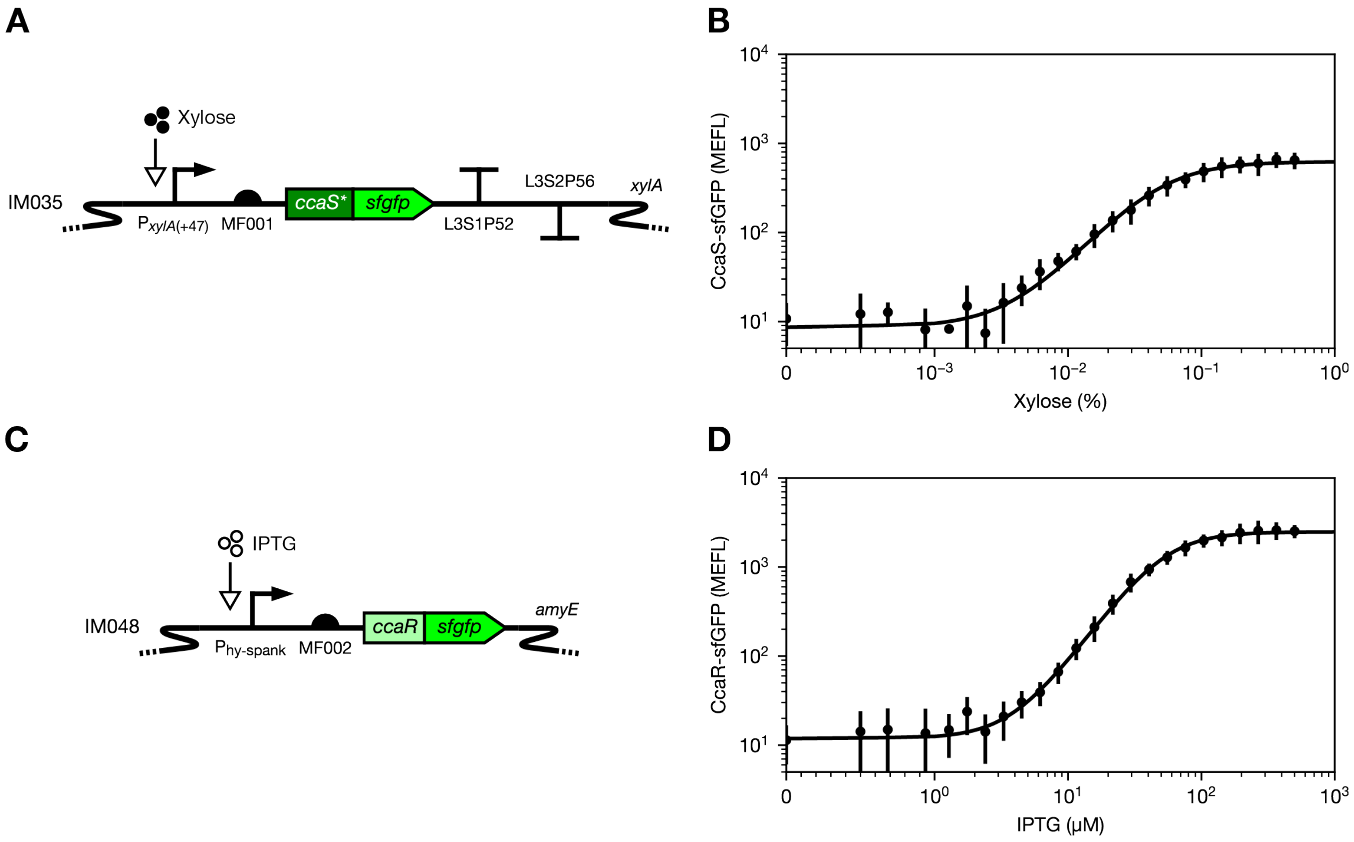

Supplementary Figure 15. Expression curves of CcaS and CcaR used to calculate inducer concentrations in Supplementary Figure 17. (A) Schematic of IM035, equivalent to LSM v0.2 with *sfgfp* fused to the C-terminus of *ccaS*, used to measure xylose-dependent CcaS expression. **(B)** Measured CcaS-sfGFP fluorescence in response to xylose. **(C)** Schematics of IM048, equivalent to TOM v0.2 with *sfgfp* fused to the C-terminus of *ccaR*, used to measure IPTG-dependent CcaR expression. **(D)** Measured CcaR-sfGFP fluorescence in response to IPTG. Markers and error bars show the mean and standard deviation of three experiments run on separate days. Black lines represent Hill Function model fits (**Methods**).

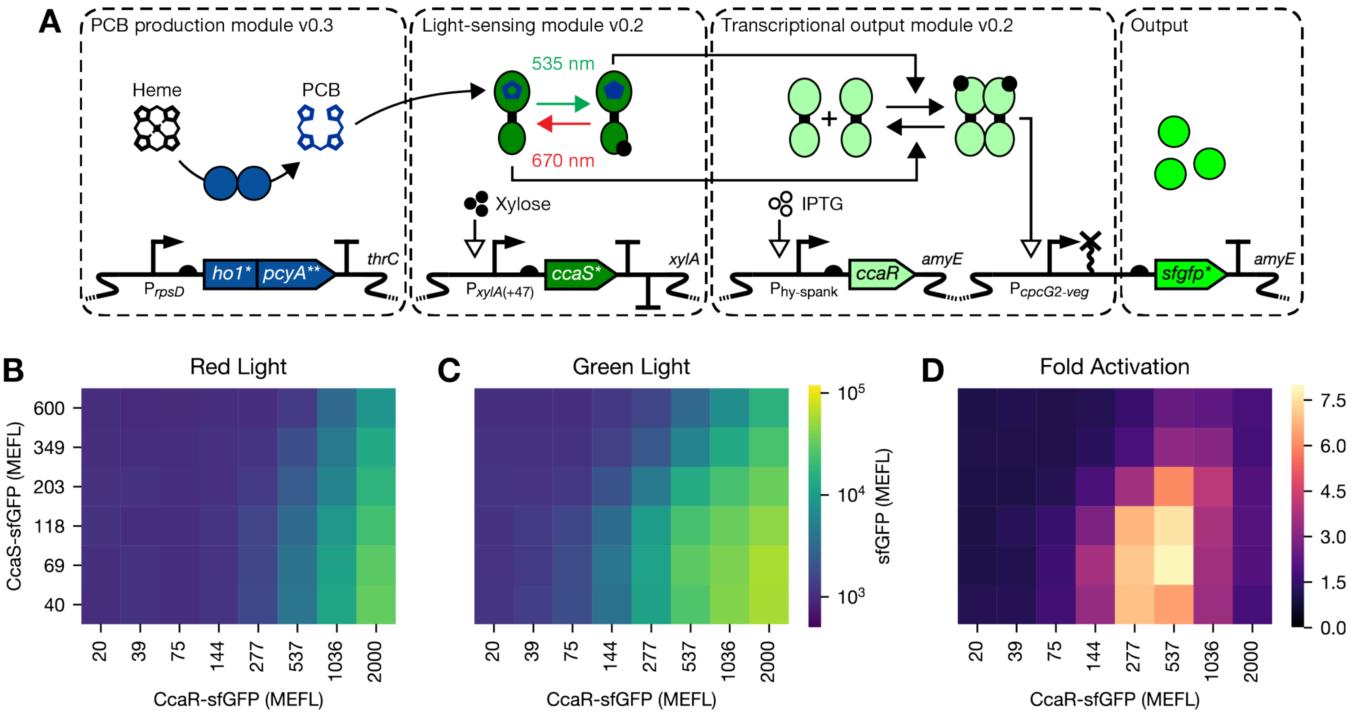

Supplementary Figure 16. Characterization of *B. subtilis* CcaSR v0.2. (A) Genetic device schematic. **(B and C)** sfGFP fluorescence of bacteria expressing CcaSR v0.2 and grown in the presence of different concentrations of IPTG and Xylose and saturating intensities of **(B)** red or **(C)** green light. IPTG and Xylose concentrations to achieve the indicated CcaS-sfGFP and CcaR-sfGFP expression levels were calculated using the dose-response curves in **Supplementary Figure 14**. **(D)** Fold increase in sfGFP fluorescence in green light compared to red light at each xylose and IPTG condition. Datapoints show the mean of three experiments run on separate days.

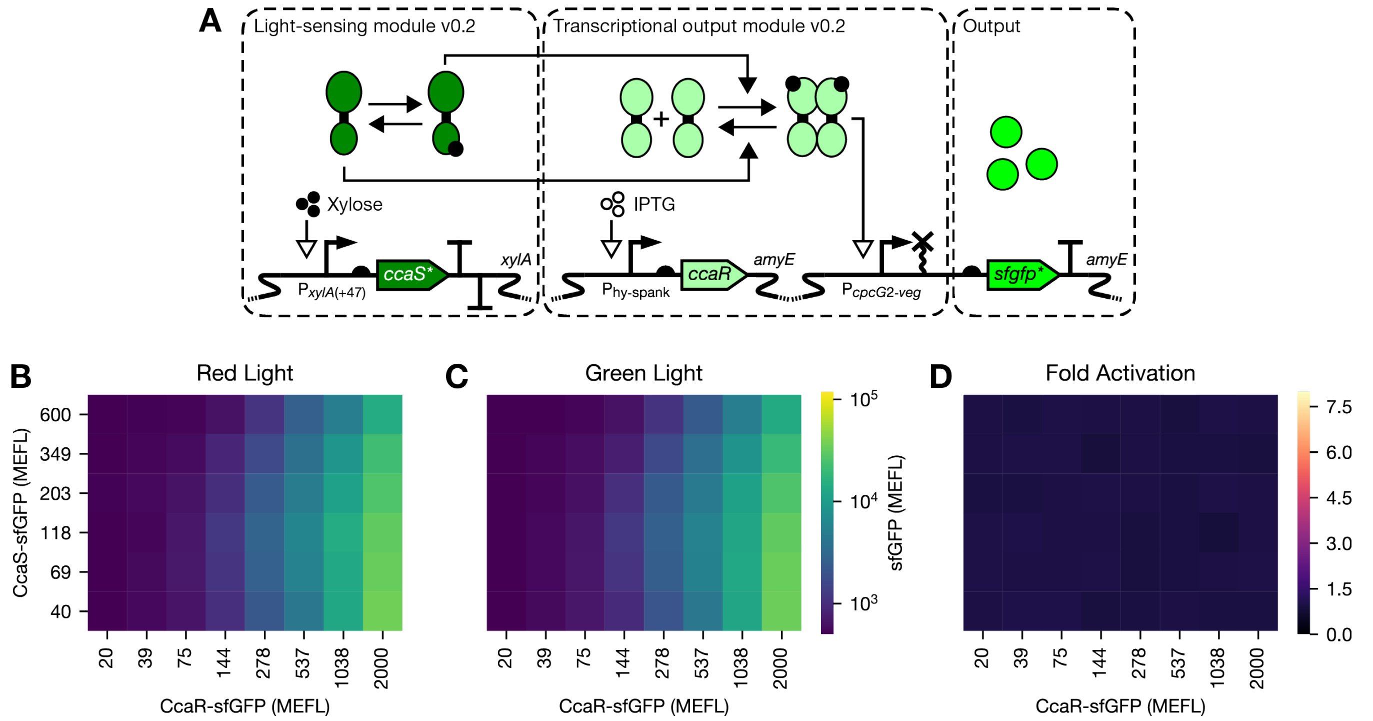

Supplementary Figure 17. Characterization of a negative control *B. subtilis* CcaSR v0.2 lacking a PPM. (A) Genetic device schematic. **(B** and **C)** sfGFP fluorescence of bacteria expressing LSM v0.2 and TOM v0.2 and grown in the presence of different concentrations of IPTG and Xylose and saturating intensities of **(B)** red or **(C)** green light. IPTG and Xylose concentrations to achieve the indicated CcaS-sfGFP and CcaR-sfGFP expression levels were calculated using the dose-response curves in **Supplementary Figure 15**. **(D)** Fold increase in sfGFP fluorescence in green light compared to red light at each xylose and IPTG condition. Datapoints show the mean of three experiments run on separate days.

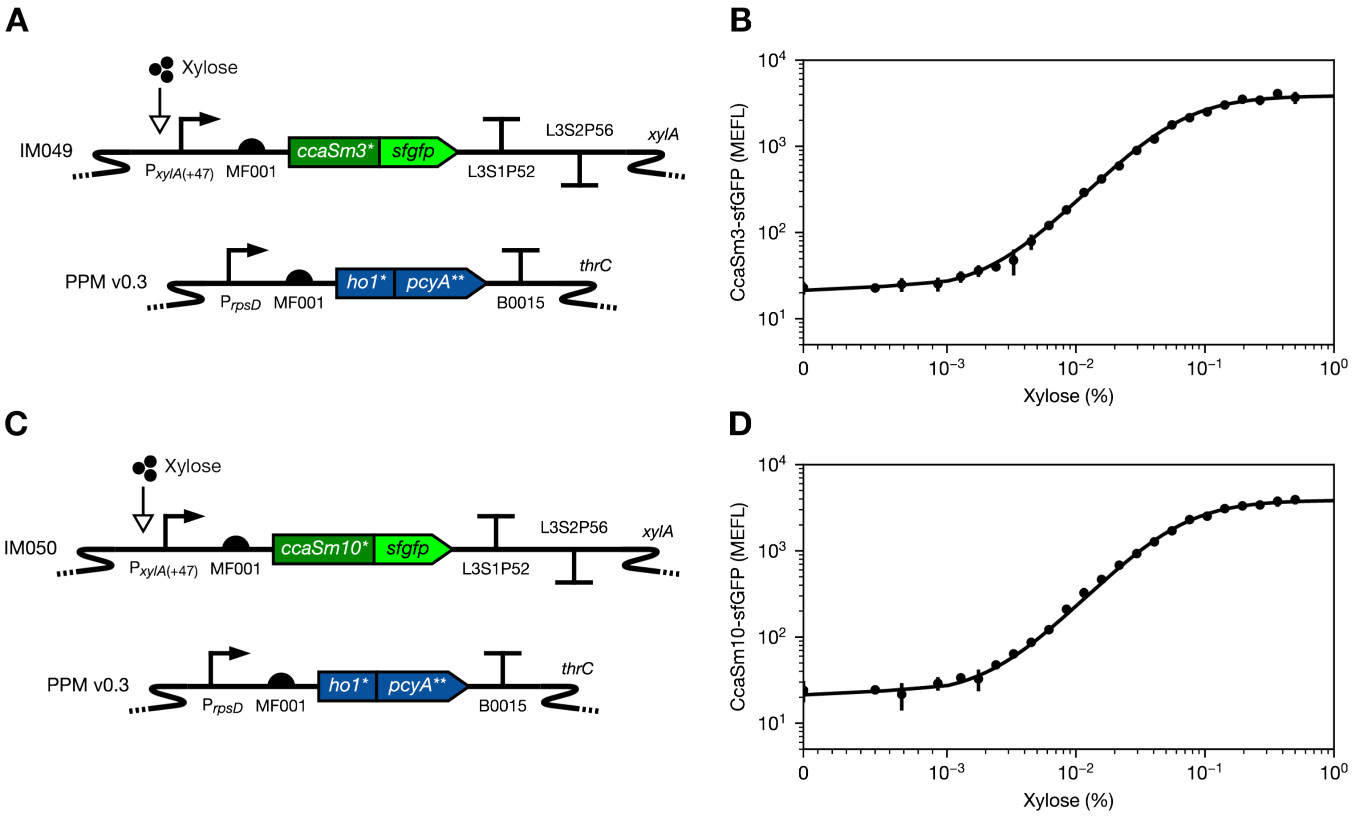

Supplementary Figure 18. Expression curves of CcaSm3 and CcaSm10 used to calculate inducer concentrations in Supplementary Figure 19 and Supplementary Figure 20. (A) Schematics of integration modules used to measure xylose-dependent CcaSm3 expression in a PCB-producing strain. IM049 is equivalent to LSM v0.3a with *sfgfp* fused to the C-terminus of *ccaSm3*. **(B)** Measured CcaSm3-sfGFP fluorescence in response to xylose. **(C)** Schematics of integration modules used to measure xylose-dependent CcaSm10 expression in a PCB-producing strain. IM050 is equivalent to LSM v0.3b with *sfgfp* fused to the C-terminus of *ccaSm10*. **(D)** Measured CcaSm10-sfGFP fluorescence in response to xylose. Markers and error bars show the mean and standard deviation of three experiments run on separate days. Black lines represent Hill Function model fits (**Methods**).

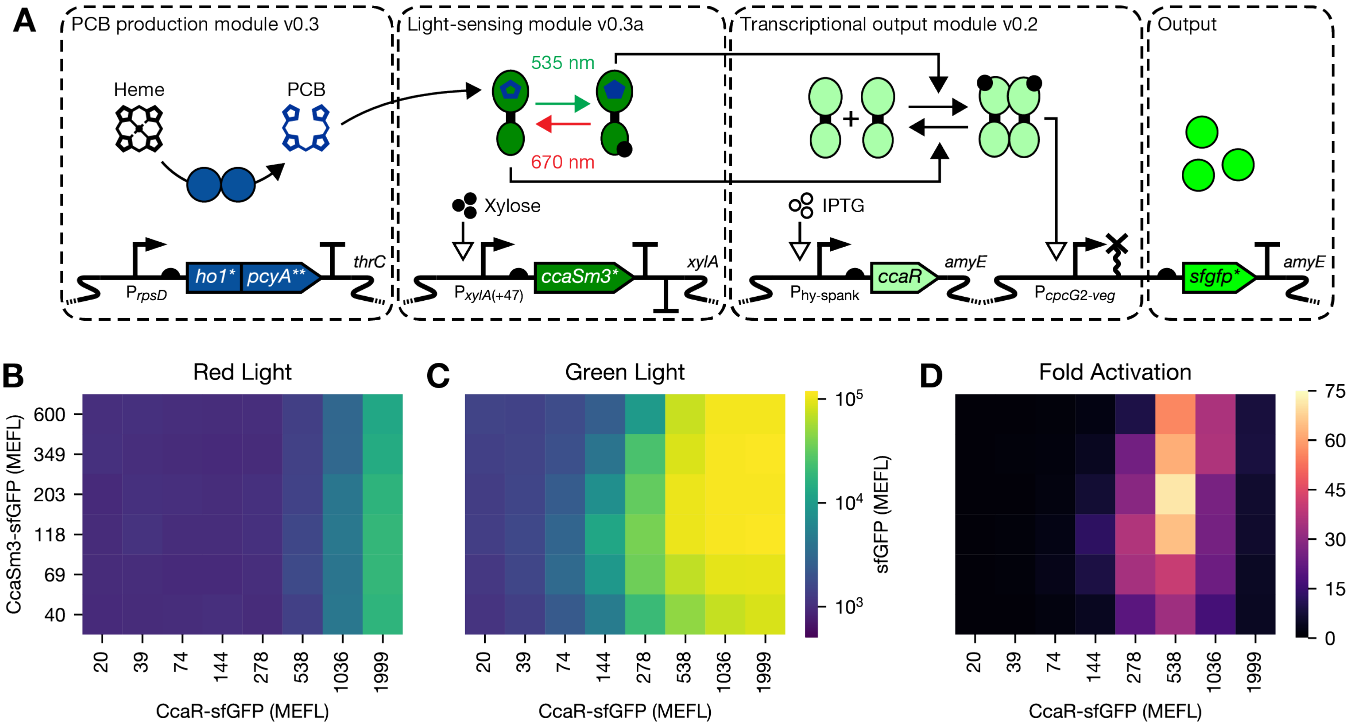

Supplementary Figure 19. Characterization of *B. subtilis* CcaSR v1.0. (A) Genetic device schematic. **(B and C)** sfGFP fluorescence of bacteria expressing CcaSR v1.0 and grown in the presence of different concentrations of IPTG and Xylose and saturating intensities of **(B)** red or **(C)** green light. IPTG and Xylose concentrations to achieve the indicated CcaSm3-sfGFP and CcaR-sfGFP expression levels were calculated using the dose-response curves in **Supplementary Figure 18** and **Supplementary Figure 14** respectively. **(D)** Fold increase in sfGFP fluorescence in green light compared to red light at each xylose and IPTG condition. Datapoints show the mean of three experiments run on separate days.

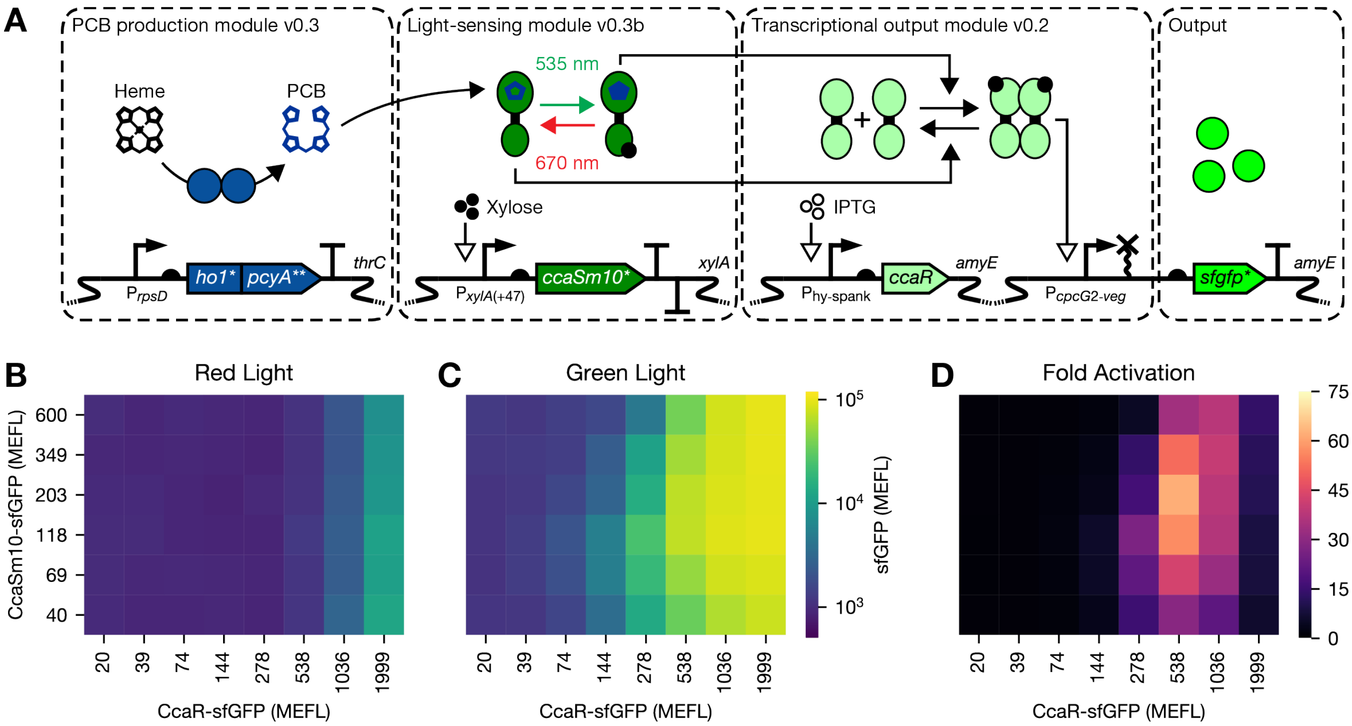

Supplementary Figure 20. Characterization of *B. subtilis* CcaSR v0.2 with CcaS replaced with CcaSm10. (A) Genetic device schematic. **(B** and **C)** sfGFP fluorescence of bacteria expressing PPM v0.3, LSM v0.3b, and TOM v0.2, and grown in the presence of different concentrations of IPTG and Xylose and saturating intensities of **(B)** red or **(C)** green light. IPTG and Xylose concentrations to achieve the indicated CcaSm10-sfGFP and CcaR-sfGFP expression levels were calculated using the dose-response curves in **Supplementary Figure 18** and **Supplementary Figure 14** respectively. **(D)** Fold increase in sfGFP fluorescence in green light compared to red light at each xylose and IPTG condition. Datapoints show the mean of three experiments run on separate days.

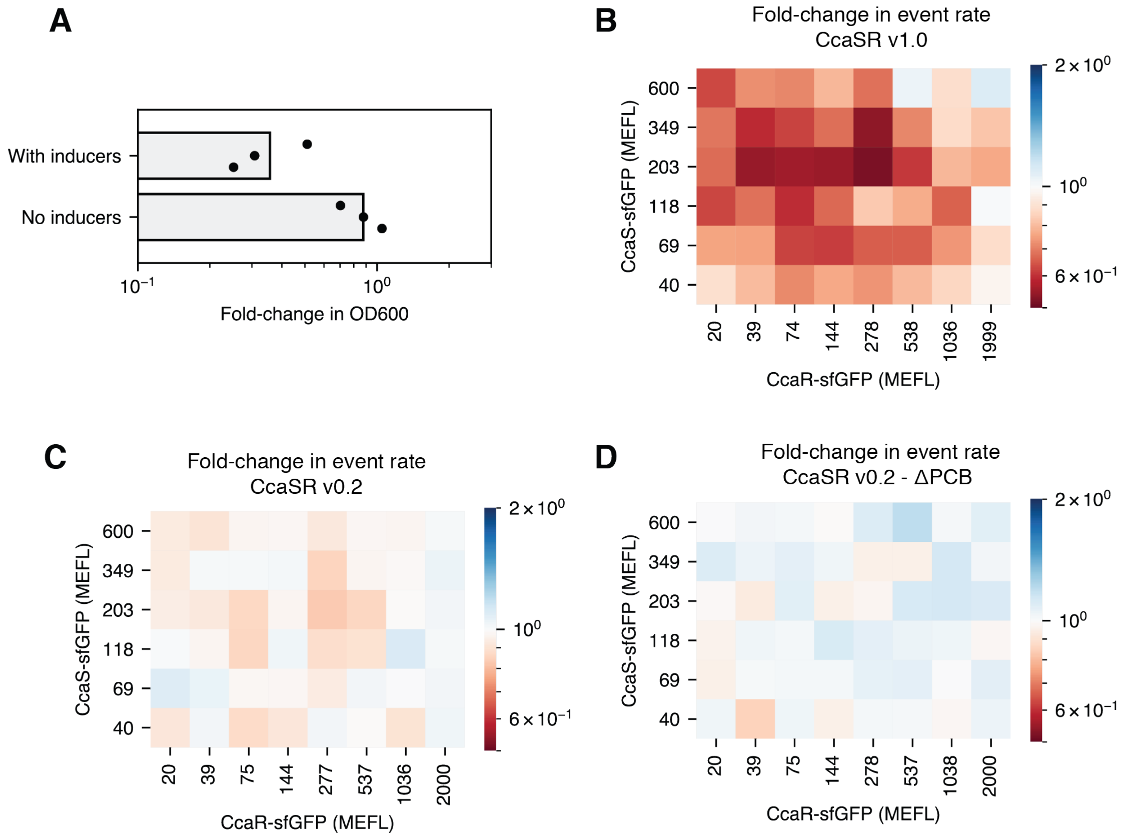

Supplementary Figure 21. Light-dependent growth changes in CcaSR v1.0 are not due to phototoxicity. (A) *B. subtilis* CcaSR v1.0 cells were grown for 12 hours under saturating amounts of green and red light, with and without the optimal amounts of inducers IPTG and xylose, after which the OD600 of each culture was measured. Dots show the ratio between the OD600 under green vs. red light in each replicate. While the final density under green light is lower when inducers are present, this effect almost disappears in the absence of inducers, suggesting that this is not due to phototoxicity. **(B)** Ratio of event rates (number of cells passing through the flow cytometer per second) under green vs. red light in the experiment shown in **Supplementary Figure 19**. Note that a region with a significant growth effect is present in the center area where the sfGFP fold-change is the highest (**Supplementary Figure 19**). We hypothesize that sfGFP production in CcaSR v1.0 is large enough to influence cell growth, thus fold-change in sfGFP output is correlated with fold-change in event rates. **(C)** Ratio of event rates under green vs. red light for CcaSR v0.2 (PPM v0.3, LSM v0.2, and TOM v0.2), where sfGFP expression levels are

overall lower than with CcaSR v1.0 (**Supplementary Figure 16**). Here, light-dependent growth effects are absent. **(D)** Ratio of event rates for the ΔPCB control of CcaSR v0.2 (LSM v0.2 and TOM v0.2) (**Supplementary Figure 17**) also fail to show light-dependent changes in final cell density.

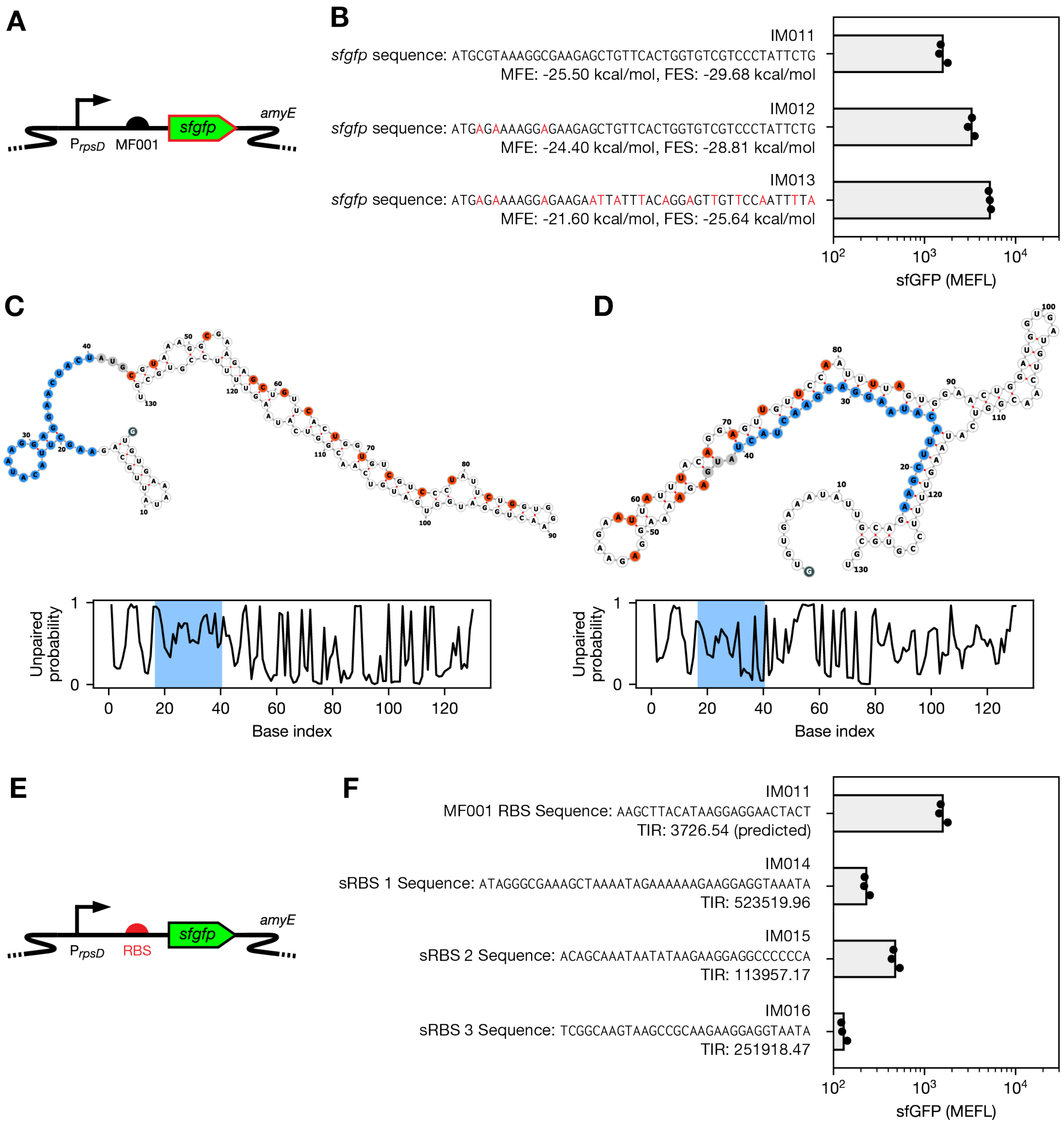

Supplementary Figure 22. Codon optimization to reduce mRNA secondary structure around the RBS can increase gene expression beyond RBS design methods. (A) Schematic of *sfgfp* expressed from the constitutive promoter P_{rpsD} and synthetic RBS MF001. **(B)** Measured fluorescence when expressing the original *sfgfp* sequence (top), or one where the initial 5 (middle) or 15 (bottom, *sfgfp**) codons were

optimized for reduced secondary structure stability. The initial ORF sequences are shown, and modifications are highlighted in red. Minimum free energy (MFE) and free energy of strand (FES) values predicted by Nupack[2] are indicated. (**C** and **D**) Secondary structure analysis of *sfgfp* and *sfgfp**. Top: MFE secondary structure of the mRNA from the 5' end to the 90th base of the *sfgfp* ORF. The 5' end is highlighted in dark gray, the RBS sequence in blue, the translation start codon ATG in light grey, and the bases that differ between the original and optimized sequences in orange. Bottom: probability of finding each base in an unpaired configuration. The RBS region is shown shaded in blue. Even though the unpaired probability of the RBS region does not increase after optimization, the probability of the initial ORF section does. This is consistent with the results of a recent study where structure in the initial ORF sequence was found to greatly affect translation as well[3] **(E)** Schematic of *sfgfp* expressed from the constitutive promoter P_{rpsD} and different synthetic RBSs. **(F)** Measured fluorescence when expressing *sfgfp* using synthetic RBS MF001, or RBSs designed by the RBS Calculator version 2.1[4], with the option to maximize the Translation Initiation Rate. Predicted Translation Initiation Rates are indicated. Bars show the mean of three experiments run on separate days. Dots show values of individual experimental replicates.

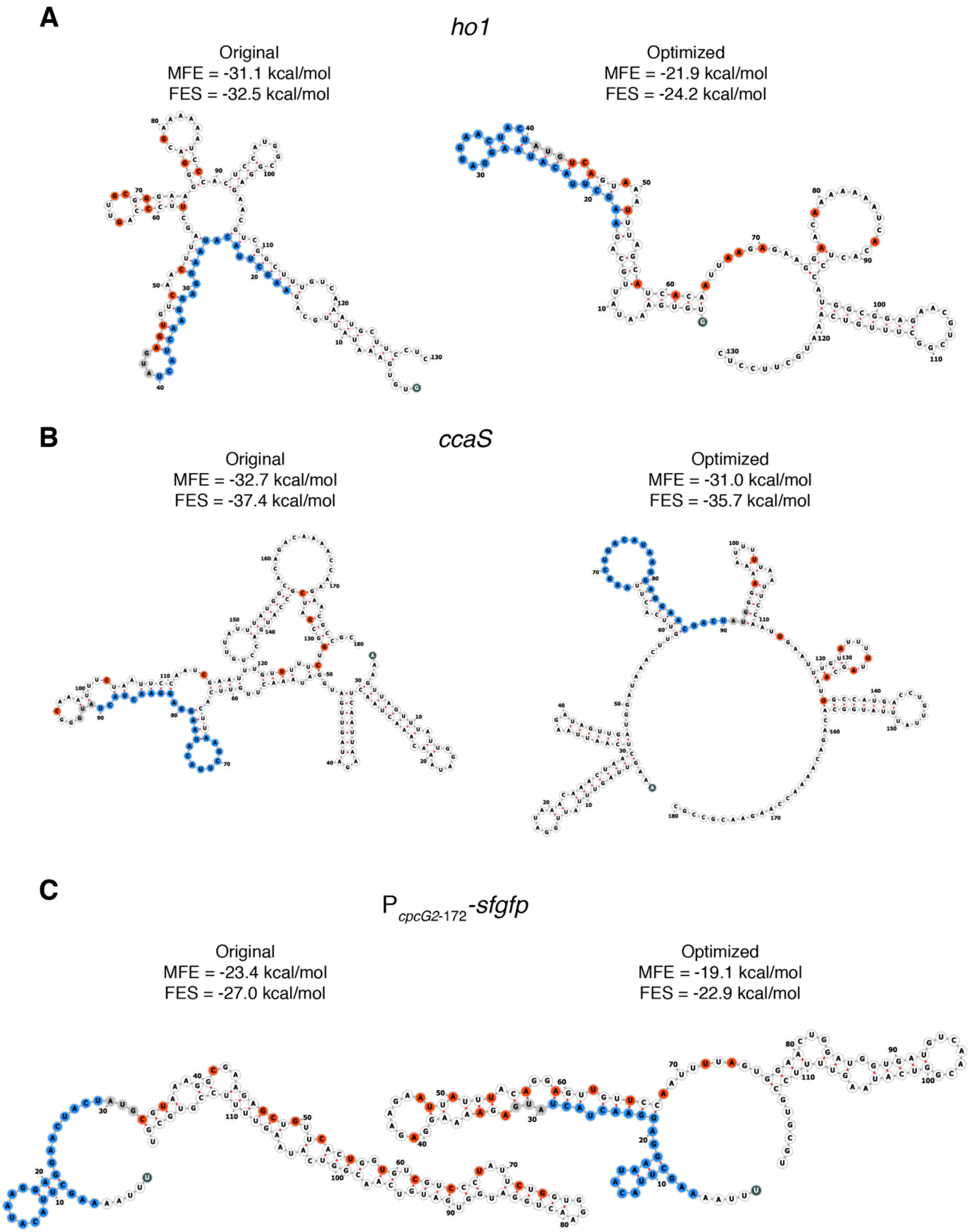

Supplementary Figure 23. Predicted Minimum Free Energy (MFE) secondary structure of ORF before and after codon optimization of their initial sequence. mRNA sequences from the +1 site to

the 90th base of the respective ORF are shown in the configuration predicted by Nupack[2] before (left) and after (right) codon optimization of the initial sequence. Forna[5] was used to generate the structure representations. Each structure depicts the 5' end of the mRNA (dark gray, white font), the RBS sequence (blue), the translation start codon (ATG in light grey) and the bases that differ between the original and optimized sequences (orange).

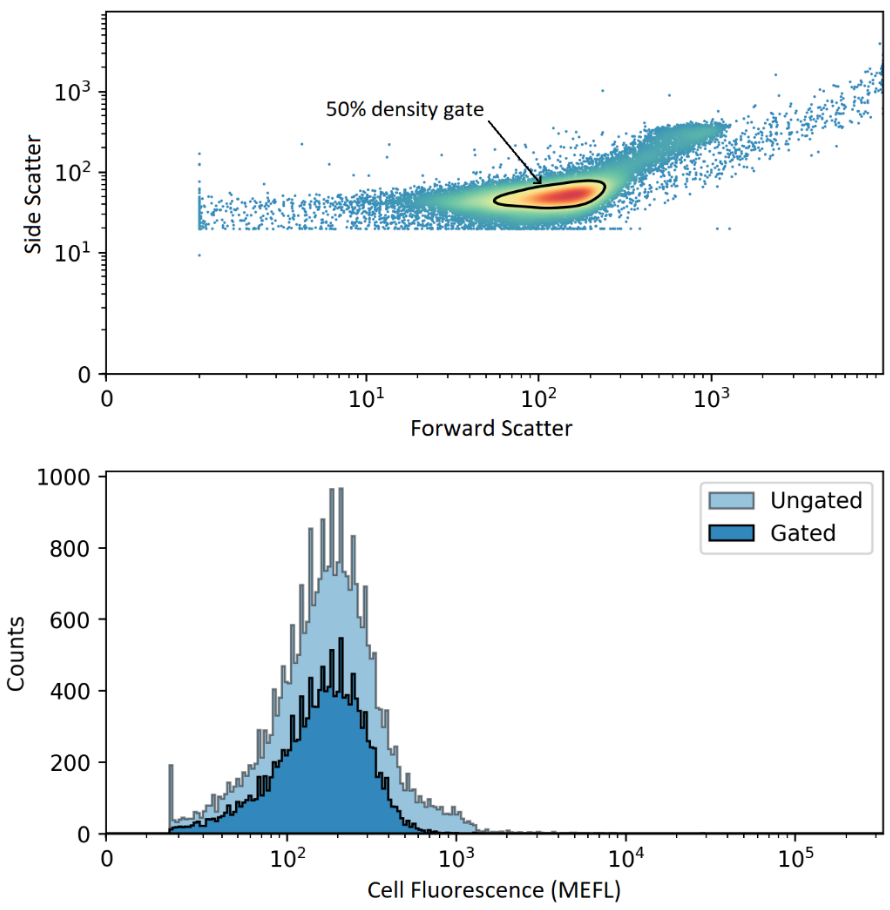

Supplementary Figure 24. **Density gating of flow cytometry data.** Top: forward/side scatter of a representative *B. subtilis* sample. Each dot represents a particle (cell, cell doublet, debris) detected by the flow cytometer. Color indicates density (number of particles with similar values of forward and side scatter). The black line represents the density gate, and surrounds the highest density area containing 50% of the particles detected by the flow cytometer. Bottom: Cell fluorescence histogram of all particles in the forward/side scatter plot above, before and after applying the density gate.

References

1. Castillo-Hair, S. M., Fujita, M., Igoshin, O. A. & Tabor, J. J. An engineered B. subtilis inducible promoter system with over 10,000-fold dynamic range. *ACS Synth. Biol.*
2. Zadeh, J. N. *et al.* NUPACK: Analysis and design of nucleic acid systems. *J. Comput. Chem.* **32**, 170–173 (2011).
3. Espah Borujeni, A. *et al.* Precise quantification of translation inhibition by mRNA structures that overlap with the ribosomal footprint in N-terminal coding sequences. *Nucleic Acids Res.* **45**, 5437–5448 (2017).
4. Espah Borujeni, A., Channarasappa, A. S. & Salis, H. M. Translation rate is controlled by coupled trade-offs between site accessibility, selective RNA unfolding and sliding at upstream standby sites. *Nucleic Acids Res.* **42**, 2646–2659 (2014).
5. Kerpedjiev, P., Hammer, S. & Hofacker, I. L. Forna (force-directed RNA): Simple and effective online RNA secondary structure diagrams. *Bioinformatics* **31**, 3377–3379 (2015).
