# Supplementary material for: Optogenetic control of Bacillus subtilis gene expression
Source: Nat Commun. 2019 Jul 15;10:3099. doi: 10.1038/s41467-019-10906-6 (PMC6629627; doi:10.1038/s41467-019-10906-6)
Supplement: Supplementary file 3 — Description of Additional Supplementary Files [file 41467_2019_10906_MOESM3_ESM.docx]

**Title:** Supplementary Dataset 1
**Description:** List of engineered strains

**Title:** Supplementary Dataset 2
**Description:** List of genetic parts

**Title:** Supplementary Dataset 3
**Description:** List of integration modules

**Title:** Supplementary Dataset 4
**Description:** Hill function fit parameters
