# Supplementary material for: Optogenetic control of Bacillus subtilis gene expression
Source: Nat Commun. 2019 Jul 15;10:3099. doi: 10.1038/s41467-019-10906-6 (PMC6629627; doi:10.1038/s41467-019-10906-6)
Supplement: Supplementary file 8 — Reporting Summary [file 41467_2019_10906_MOESM8_ESM.pdf]

nature research

Corresponding author(s): Jeffrey J. Tabor

Last updated by author(s): 2019/05/31

Reporting Summary

Nature Research wishes to improve the reproducibility of the work that we publish. This form provides structure for consistency and transparency in reporting. For further information on Nature Research policies, see Authors & Referees and the Editorial Policy Checklist.

Statistics

For all statistical analyses, confirm that the following items are present in the figure legend, table legend, main text, or Methods section.

n/a	Confirmed	
☐	☒	The exact sample size (*n*) for each experimental group/condition, given as a discrete number and unit of measurement
☐	☒	A statement on whether measurements were taken from distinct samples or whether the same sample was measured repeatedly
☐	☒	The statistical test(s) used AND whether they are one- or two-sided<br>*Only common tests should be described solely by name; describe more complex techniques in the Methods section.*
☒	☐	A description of all covariates tested
☒	☐	A description of any assumptions or corrections, such as tests of normality and adjustment for multiple comparisons
☐	☒	A full description of the statistical parameters including central tendency (e.g. means) or other basic estimates (e.g. regression coefficient) AND variation (e.g. standard deviation) or associated estimates of uncertainty (e.g. confidence intervals)
☐	☒	For null hypothesis testing, the test statistic (e.g. *F*, *t*, *r*) with confidence intervals, effect sizes, degrees of freedom and *P* value noted<br>*Give P values as exact values whenever suitable.*
☒	☐	For Bayesian analysis, information on the choice of priors and Markov chain Monte Carlo settings
☒	☐	For hierarchical and complex designs, identification of the appropriate level for tests and full reporting of outcomes
☒	☐	Estimates of effect sizes (e.g. Cohen's *d*, Pearson's *r*), indicating how they were calculated

Our web collection on statistics for biologists contains articles on many of the points above.

Software and code

Policy information about availability of computer code

Data collection	FlowJo CE (v7.5.110.7) software was used to interface with the flow cytometer during data collection.
Data analysis	Flow cytometry sample data was processed and analyzed using FlowCal (v1.2.0) software. Processed cytometry data was further analyzed and plotted using the Python 3.6 packages Numpy (v1.14.2), Scipy (v0.19.1), Matplotlib (v2.1.0), Pandas (0.20.3), and Seaborn (0.8). Transfer function fits were generated by Python 3.6 package Lmfit (v0.9.7). Kinetic simulations were performed with Python 3.6 package pydde (0.2.2). mRNA secondary structure analysis was performed using Python 2.7 package multistrand (2.1).

For manuscripts utilizing custom algorithms or software that are central to the research but not yet described in published literature, software must be made available to editors/reviewers. We strongly encourage code deposition in a community repository (e.g. GitHub). See the Nature Research guidelines for submitting code & software for further information.

Data

Policy information about availability of data

All manuscripts must include a data availability statement. This statement should provide the following information, where applicable:

- Accession codes, unique identifiers, or web links for publicly available datasets
- A list of figures that have associated raw data
- A description of any restrictions on data availability

Sequences of all integration modules used in this study are available from Genbank via the accession numbers listed in Supplementary Data 3. Flow cytometry data and scripts used to generate all figures are available from figshare (DOI: 10.6084/m9.figshare.8198999). Parameters of Hill function fits can be found in Supplementary Data 4.

Field-specific reporting

Please select the one below that is the best fit for your research. If you are not sure, read the appropriate sections before making your selection.

☒ Life sciences ☐ Behavioural & social sciences ☐ Ecological, evolutionary & environmental sciences

For a reference copy of the document with all sections, see nature.com/documents/nr-reporting-summary-flat.pdf

Life sciences study design

All studies must disclose on these points even when the disclosure is negative.

Sample size	Sample size (3 independent bacterial populations of ~10,000 or more bacteria measured by flow cytometry on separate days) was determined based upon literature standards, our extensive prior experience with these types of data, and analysis of the current data, which revealed that all reported results are highly reproducible.
Data exclusions	No data were excluded from analysis.
Replication	All experiments were repeated 3 times. All attempts to replicate were successful.
Randomization	Inducer concentrations and light conditions were randomized across wells in 24 well plates used in the experiments in Figures 5, 6, and Supplementary Figures 1, and 14 to 21. No randomization was performed for the remaining data.
Blinding	Blinding was not relevant to this study since samples were not grouped.

Reporting for specific materials, systems and methods

We require information from authors about some types of materials, experimental systems and methods used in many studies. Here, indicate whether each material, system or method listed is relevant to your study. If you are not sure if a list item applies to your research, read the appropriate section before selecting a response.

Materials & experimental systems

n/a	Involved in the study
☒	☐ Antibodies
☒	☐ Eukaryotic cell lines
☒	☐ Palaeontology
☒	☐ Animals and other organisms
☒	☐ Human research participants
☒	☐ Clinical data

Methods

n/a	Involved in the study
☒	☐ ChIP-seq
☐	☒ Flow cytometry
☒	☐ MRI-based neuroimaging

Flow Cytometry

Plots

Confirm that:

☒ The axis labels state the marker and fluorochrome used (e.g. CD4-FITC).

☒ The axis scales are clearly visible. Include numbers along axes only for bottom left plot of group (a 'group' is an analysis of identical markers).

☐ All plots are contour plots with outliers or pseudocolor plots.

☒ A numerical value for number of cells or percentage (with statistics) is provided.

Methodology

Sample preparation	Cell samples were transferred to an ice water bath to arrest growth. 100 µL of each sample was transferred to a flow cytometry tube containing 1 mL phosphate buffered saline (PBS) for measurement.
Instrument	sfGFP fluorescence measurements were performed using a BD FACScan (BD, Franklin Lakes, NJ, U.S.A) with custom blue (488 nm, 30 mW) and yellow (561 nm, 50 mW) solid-state lasers (Cytek Biosciences) and a custom 510/21 nm FL1 (sfGFP) acquisition channel emission filter. Cph1(Y176H) fluorescence measurements were performed using a BD FACSCanto II. The fluorescent channel used (APC-A) used a 633 nm red laser as the excitation source, and an emission window of 670/50 nm.
Software	FlowJo CE (v7.5.110.7) software was used to interface with the flow cytometer and acquire sample data. All samples were processed and analyzed using FlowCal (v1.2.0).

Cell population abundance	Sample acquisition was performed at 500 – 2,000 event/s. 10,000 to 30,000 events were collected and stored for each culture sample.
Gating strategy	Events detected by the cytometer in the bottom 32% of the SSC detection range (32% threshold) were not recorded to reduce contamination from non-bacterial events. The first 250 and final 100 recorded events were removed from all samples (fluidic transients). Events with values in the first or last bin of the FSC, SSC, or FL1 (bacterial only) were removed as these values exceed the limits of detection. A final density gate is applied to identify the non-aggregate fluorescence calibration bead (30% of events retained) and bacterial cell populations (50% of events retained) used in this study.

☒ Tick this box to confirm that a figure exemplifying the gating strategy is provided in the Supplementary Information.
